# A Bibliometric and Systematic Review of the Use of Recycled Composite Materials with an Emphasis on the Mechanical Performance of Structures

**DOI:** 10.3390/ma18030607

**Published:** 2025-01-29

**Authors:** Cristina Veres, Maria Tănase

**Affiliations:** 1 Department of Industrial Engineering and Management, George Emil Palade University of Medicine, Pharmacy, Science, and Technology of Targu-Mures, Nicolae Iorga Street, 1, 540088 Targu Mures, Romania; cristina.veres@umfst.ro; 2 Doctoral School of I.O.S.U.D., George Emil Palade University of Medicine, Pharmacy, Science and Technology of Targu Mures, Gheorghe Marinescu Street, 38, 540142 Targu Mures, Romania; 3 Mechanical Engineering Department, Petroleum-Gas University of Ploiesti, 100680 Ploiesti, Romania

**Keywords:** composite materials, fiber-reinforced polymers, recycled, advanced recycling technologies, hybrid composites, mechanical properties, bibliometric analysis, VOSviewer

## Abstract

The paper provides a bibliometric and systematic review of the utilization of recycled composite materials, focusing on their mechanical performance in structural applications. Analyzing 1525 publications from the Web of Science database using VOSviewer, the study identifies trends, key topics, and collaboration networks. Findings show that recycled fiber-reinforced polymers (FRPs) maintain up to 93% of their virgin tensile strength under optimal pyrolysis conditions, while mechanical recycling can reduce tensile properties by up to 29%. Advances in hybrid composites and recycling technologies have facilitated sustainable applications in the construction, transportation, and energy sectors. China is a dominant contributor to research on recycled composites, with the USA making strong contributions as well. Other countries, including England, India, and Germany, have active research communities, while nations such as Saudi Arabia, the Republic of Korea, and Spain demonstrate growing global participation in this field. The bibliometric analysis highlights a significant increase in global research activity, with key trends focusing on additive manufacturing, circular economy practices, and sustainability. Despite these advancements, challenges persist, including high operational costs and variability in recycled material quality. This review underscores the need for standardized protocols and improved lifecycle assessments to accelerate the adoption of recycled composites in engineering practices.

## 1. Introduction

Composite materials have emerged as a cornerstone of modern engineering, offering unparalleled advantages in terms of strength-to-weight ratio, durability, and design flexibility. These materials, composed of two or more constituent materials with distinct physical or chemical properties, are engineered to achieve superior performance across a wide range of applications [1,2,3,4]. Among them, fiber-reinforced polymers (FRPs) have gained significant prominence due to their exceptional mechanical and structural properties [5,6,7,8,9]. FRPs, which are composed of a polymer matrix reinforced with fibers such as glass, carbon, or aramid, are extensively used in sectors such as the construction, transportation, energy, and marine industries [10,11,12,13]. The composites industry has demonstrated long-term growth since the 1960s, reaching 11.8 million tons in 2019 [14]. The global composites market size was USD 99.91 billion in 2019 and is projected to reach USD 112.0 billion by 2027 [15].

In transportation, the use of FRPs in automotive and aerospace manufacturing is growing rapidly, driven by the demand for lightweight, high-strength materials. The transportation composites market is estimated to develop at an impressive 12.7% compound annual growth rate (CAGR) this year [16].

In the energy sector, particularly in wind turbine manufacturing, FRPs account for a significant portion of the global production [17]. Similarly, the marine industry has also embraced FRPs, particularly in boat- and shipbuilding, where their strength-to-weight ratio makes them an ideal choice [17].

In the construction industry, FRPs are increasingly utilized in structural applications such as bridges, beams, and reinforcement systems for concrete structures. Their high corrosion resistance and lightweight properties make them ideal for replacing traditional materials like steel, especially in harsh environments where durability is critical [18,19,20,21,22,23,24,25,26,27,28]. Several studies have focused on the tensile and mechanical properties of fiber-reinforced polymer (FRP) bars under varying conditions. Almusallam et al. [26] explored the degradation of tensile properties of glass fiber-reinforced polymer (GFRP) bars embedded in concrete under severe laboratory and field environmental conditions. Similarly, Ashrafi et al. [29] investigated the effects of mechanical and thermal properties of FRP bars on their tensile performance at elevated temperatures, highlighting the critical role of temperature in material performance. Wang et al. [27] also conducted an experimental study on the mechanical properties of both FRP and steel reinforcing bars at elevated temperatures. Robert et al. [30] examined the long-term durability of GFRP reinforcing bars embedded in moist concrete, revealing critical insights into moisture-induced degradation. Jia et al. [31] provided experimental findings on the durability of GFRP bars under various environmental conditions, offering practical implications for infrastructure applications.

The use of FRPs in bridge construction, for example, has demonstrated significant benefits in terms of reduced maintenance costs and extended service life. Similarly, in the transportation sector, FRPs are used in the manufacture of lightweight vehicle components and railway infrastructure, contributing to improved fuel efficiency and reduced environmental impact [32,33,34].

The energy sector also benefits significantly from the application of FRPs. Wind turbine blades, for instance, are predominantly constructed from fiber-reinforced polymers due to their excellent stiffness and fatigue resistance [35,36,37]. The offshore oil and gas industry utilizes FRP components for risers, pipelines, and storage tanks, where resistance to corrosion and marine environments is essential [6,38,39,40,41]. In the marine industry, FRPs are widely used in shipbuilding for hulls and decks, offering reduced weight and enhanced performance under dynamic loading conditions [42,43].

The increasing production and use of FRP materials have necessitated a shift toward sustainable practices, particularly in their recycling and reuse. Recycling composites aligns with the principles of the circular economy, which emphasizes the reduction of waste and the maximization of resource efficiency. The transition to sustainable composite use is driven by growing regulatory pressures, environmental concerns, and the need for cost-effective raw material alternatives [44,45]. As FRPs become more prevalent in structural applications, addressing their end-of-life management has become a critical challenge for both industry and academia.

Recycling technologies for FRPs have evolved significantly over recent years, with mechanical, thermal, and chemical recycling methods being the most prominent [18,46,47]. Mechanical recycling involves grinding composite materials into smaller particles for reuse, while thermal recycling focuses on energy recovery through controlled combustion. Chemical recycling, on the other hand, enables the recovery of high-quality fibers and resins through processes such as solvolysis and pyrolysis. Each method offers unique advantages and challenges in terms of scalability, efficiency, and environmental impact.

Structural applications of recycled composites are becoming increasingly viable due to advancements in recycling technologies. For example, recycled glass fibers have been successfully integrated into secondary structural components such as concrete, asphalt, and various composite materials, contributing to sustainability and performance enhancement [10,34,48,49,50,51]. Moreover, the development of hybrid composites, which combine recycled and virgin materials, has opened new possibilities for creating sustainable and high-performance structural systems [52,53,54].

Recent advancements in the use of recycled composites have also highlighted the potential of plant fiber-based materials for structural applications. Plant fibers, with their high cellulose content, exhibit strong interfacial adhesion with polymer matrices, which enhances the mechanical properties of the resulting composites. For instance, Marcuello et al. [55] highlighted the importance of fiber–matrix interactions in bio-based composites, demonstrating that hypolignified plant fibers form stronger nanoscale bonds with poly (butylene succinate) (PBS) than with lignified fibers. Furthermore, Janowski et al. [56] investigated the properties of 3-hydroxybutyrate-co-3-hydroxyvalerate (PHBV)-based biocomposites reinforced with ground buckwheat hulls, demonstrating their potential for sustainable structural applications. The study revealed that buckwheat hulls improve dimensional stability and stiffness while reducing material shrinkage. Although initial recycling cycles slightly decreased mechanical properties, subsequent cycles stabilized these characteristics.

Incorporating recycled composites into structural applications is a critical step toward achieving sustainability in engineering and manufacturing. The adoption of lifecycle assessment (LCA) methodologies has highlighted the environmental benefits of recycling composites, including reduced carbon emissions, lower energy consumption, and decreased dependence on virgin raw materials [11,12,33]. Furthermore, the economic viability of recycling has been bolstered by innovations in material recovery and processing techniques, making it a practical alternative for industries seeking to reduce costs and environmental footprints [46].

Despite these advancements, challenges remain in scaling up recycling technologies to industrial levels. Issues such as variability in material quality, high processing costs, and the lack of standardized recycling protocols hinder broader adoption [18,46,57].

This paper distinguishes itself by offering a comprehensive integration of bibliometric and systematic analyses to investigate the advancements and challenges in the use of recycled FRPs in structural applications. Unlike previous studies that focus primarily on either mechanical performance or recycling technologies, this work uniquely synthesizes trends from 1525 publications, identifies key thematic areas such as sustainability and mechanical optimization, and highlights emerging technologies like additive manufacturing and hybrid composites. By combining detailed quantitative insights with practical implications, the paper provides a novel framework for understanding the global research landscape and its progression. Additionally, the inclusion of advanced bibliometric tools such as VOSviewer to map collaboration networks and keyword density adds a new perspective, offering a roadmap for future research directions and promoting the adoption of recycled composites in sustainable engineering.

## 2. Current Scenario from the Study

### 2.1. FRP Recycling Technologies

**Figure 1:**
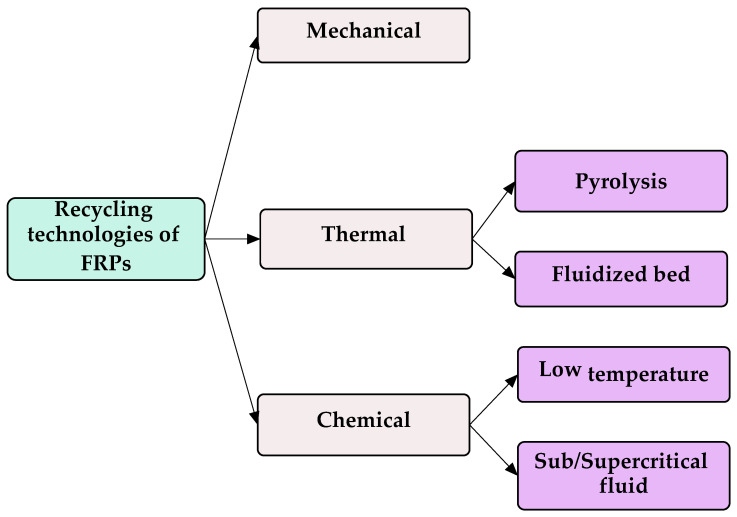
Classification of FRP recycling technologies.

Details on FRP recycling technologies (see Figure 1) are presented in Table 1.

Mechanical recycling is efficient and scalable, requiring relatively inexpensive equipment and minimal skilled labor; however, it produces low-value recycled materials that lack competitiveness with virgin materials and does not recover individual fibers [46]. In contrast, thermal recycling, particularly through pyrolysis, allows for the recovery of energy-rich by-products and is scalable for commercial applications, yet it often results in low-quality reclaimed fibers due to high temperatures, leading to a loss in fiber strength and economic viability concerns [46]. Chemical recycling offers the advantage of recovering clean fibers and reusable resin using low-risk solvents, but it is hindered by low efficiency, high costs, and significant energy consumption, alongside potential human health impacts from greenhouse gas emissions [46]. Thus, while mechanical recycling prioritizes efficiency and cost-effectiveness, thermal and chemical recycling focus on material recovery and quality, even though with varying degrees of economic and environmental feasibility.

### 2.2. Mechanical Behavior of Recycled FRP Materials

In Table 2, a comprehensive overview of various recycling methods for FRP materials is presented. The table includes the recycling method and a summary of the effects on mechanical properties, such as tensile strength, flexural strength, and modulus, found in different research studies. These details provide a clear comparison of how different recycling techniques impact the performance of the recycled fibers, highlighting improvements or reductions in specific mechanical characteristics. This table serves as a valuable resource for understanding these trade-offs.

Based on the data provided in Table 2, it can be observed that mechanical recycling often leads to reduced tensile strength (up to 29%), Young’s modulus (up to 23%), and flexural strength (up to 28%). However, methods involving carbon powder wastes (CPW) show improvements, with an up to 30% increase in flexural strength and 28% in impact strength. Thermal recycling methods, particularly at higher temperatures like 500 °C, retain up to 93% of tensile strength, but lower temperatures (350 °C, 600 °C) can cause significant reductions in mechanical properties (of up to 74%). Chemical recycling methods generally cause reductions in mechanical properties, especially at higher hydrolysis temperatures (e.g., 350 °C, reducing properties by 60%), though some processes, like sizing removal, maintain tensile strength close to virgin levels [77]. Overall, recycling methods offer varying impacts, with some improving specific properties and others reducing mechanical performance.

### 2.3. Environmental and Economic Implications

Recycling composites, particularly fiber-reinforced polymers (FRPs), have emerged as a critical strategy for addressing environmental and economic challenges. The process not only reduces the accumulation of composite waste in landfills but also conserves valuable raw materials such as fibers and resins. These benefits are especially relevant given the growing demand for sustainable practices across industries such as automotive, aerospace, and construction. One of the primary environmental benefits of recycling FRPs is the reduction in waste volume. The landfill disposal of composites poses significant challenges due to their durability and resistance to degradation. Recycling diminishes these issues by enabling the recovery and reuse of fibers and other components. For instance, mechanical recycling processes produce filler materials for concrete or new composite formulations, thereby reducing dependency on virgin resources [57,65,91].

Thermal recycling methods, such as catalytic pyrolysis, oxidation, and fluidized bed techniques, are energy-efficient and offer reasonable economic profitability. However, electrochemical methods demand significantly higher electricity input to achieve comparable profits [12].

In terms of environmental impact, solvolysis and electrochemical recycling exhibit the lowest CO_2_ emissions, making them favorable for reducing global warming potential. Conversely, landfill, incineration, and fluidized bed processes fail to meet CO_2_ reduction expectations and may exacerbate global warming concerns.

Recycled composite materials offer significant environmental benefits by reducing the demand for virgin resources, but they also present potential risks that require careful consideration. One major concern is the release of harmful substances during their lifecycle. For example, as these materials degrade over time, they can break down into microplastics, which persist in ecosystems and have been shown to accumulate in the food chain [92]. Furthermore, residual chemical additives from the original materials may leach into surrounding soil or water, posing additional threats to aquatic and terrestrial life [93].

Another important aspect to consider is the environmental cost of recycling processes themselves. Although recycling reduces waste, the methods used can be energy-intensive and contribute to greenhouse gas emissions. Mechanical recycling, which involves shredding and reprocessing, often consumes large amounts of energy, while chemical recycling relies on solvents and heat that may result in further environmental impacts [46].

On the other hand, the end-of-life challenges associated with recycled composite materials should not be overlooked. While these materials are initially diverted from landfills, their eventual disposal or re-recycling may pose difficulties, particularly if they are composed of complex or poorly degradable components [94].

From an economic perspective, the recycling of composites presents opportunities for cost savings and value creation. For instance, pyrolysis can yield high-quality fibers that retain much of their original strength, making them suitable for secondary applications at a lower cost [11,95].

The economic aspects of recycling FRP remain an important barrier to widespread adoption despite increasing environmental and regulatory pressure to recycle these materials [59]. Recycling FRPs through mechanical, thermal, or chemical methods presents inherent economic challenges, such as high operational and capital costs, limited plant capacities, and difficulties in achieving high-quality fiber recovery. The study [96] examines the environmental and financial performance of three CFRP waste treatment methods, namely landfilling, incineration, and mechanical recycling, and highlights their trade-offs. Landfilling remains a common method for end-of-life waste management due to its cost-effectiveness and low technical demands, but, due to its negative environmental and health impacts, it is the least favored option in the European Union Waste Hierarchy and should be minimized [97,98,99,100]. Even with measures like bottom sealing in place, landfilling still presents significant risks, including the contamination of water resources. Incineration, although compliant with regulations and capable of reducing primary energy consumption by generating electricity and heat, is a significant source of greenhouse gas (GHG)emissions. Mechanical recycling reduces GHG emissions and landfill waste but faces financial challenges due to high costs and low revenues when recycled carbon fibers (rCFs) replace glass fibers. To improve both environmental and financial outcomes, rCF must target higher-value applications, though property degradation during recycling limits its use to lower-value products. Emerging technologies like fluidized bed and pyrolysis-based recycling show promise, but their higher energy demands and capital costs require thorough life-cycle assessments to fully understand their trade-offs. Additionally, Hagnell and Åkermo [101] developed a recyclate value model linking the mechanical performance of recycled fibers in fiber-reinforced composites to their economic potential. The model shows that recycling carbon- and glass-reinforced thermosets can reduce material costs by up to 50% while maintaining comparable performance. This cost reduction offers designers new options for lightweight, lower-cost, and diverse stiffness designs. The proposed recycling hierarchy emphasizes the need for improved sorting and recycling techniques to enhance sustainability, open new application areas, and maximize the value of recycled materials throughout their life cycle.

Recent studies have proposed various cost modeling frameworks to address these economic hurdles. Shehab et al. [102] developed a fuzzy logic-based system for recycling cost estimation, which accounts for uncertainties in end-of-life material characteristics, transportation, and operation costs. By incorporating 243 fuzzy rules and enabling scenario-based analyses, this system provides a practical tool for decision-making in selecting cost-effective recycling methods.

Similarly, a knowledge-based system introduced by Shehab et al. [103] integrates the Technique for Order Preference by Similarity to Ideal Solution (TOPSIS) optimization method to recommend suitable recycling techniques. This approach considers factors such as material type, dismantling costs, and industry-specific requirements, providing early-stage designers and stakeholders with actionable insights into recycling costs without requiring in-depth expertise.

The adoption of recycled composite materials is significantly influenced by social and political factors. Politically, regulatory frameworks play a very important role; for instance, the European Union’s Directive on Packaging and Packaging Waste (94/62/EC) mandates that, by 2025, a minimum of 50% of all plastic packaging waste must be recycled, increasing to at least 55% by 2030 [104]. Such legislation stimulates the utilization of recycled materials by setting clear targets and encouraging compliance through policy measures. Socially, public awareness and consumer behavior are fundamental. Studies have shown that consumer engagement in recycling is influenced by macroenvironmental factors, situational contexts, and individual attitudes [105]. Enhanced public awareness campaigns and educational initiatives can drive demand for products made from recycled composites, thereby promoting sustainable practices. However, challenges remain, including resistance to change within industries and limited funding for research into recycled construction materials [106]. Additionally, the lack of standardization in the quality and performance of recycled composites can deter their widespread adoption, calling for governmental and institutional collaboration to establish universally recognized standards [106]. Furthermore, geopolitical considerations may play a role. Countries with strong recycling infrastructures and progressive environmental policies are better positioned to embrace recycled composite materials than those lacking such systems. Global cooperation and knowledge sharing can help bridge these gaps, fostering a more inclusive approach to sustainable materials. Addressing these social and political dimensions is essential for the broader adoption of recycled composite materials.

Overall, the integration of recycling into the lifecycle of composites aligns with broader sustainability goals, reducing environmental impact while enhancing economic viability. As regulations and consumer expectations continue to prioritize sustainability, the importance of composite recycling is expected to grow, driving further advancements in technologies and applications.

While advancements in recycling technologies have addressed several technical and environmental challenges, the role of standards and regulations in guiding the adoption of recycled composite materials cannot be overlooked. Standards and regulations governing the processing and use of recycled composite materials vary significantly across regions, influencing industry practices and sustainability efforts. For instance, the International Organization for Standardization (ISO) provides guidelines for the recovery and recycling of plastics, emphasizing the need for proper process monitoring and control procedures during mechanical recycling [107]. In the United States, the Environmental Protection Agency (EPA) outlines regulatory exclusions and alternative standards for recycling materials, including composites, to ensure environmental safety and promote recycling initiatives [108]. Another key standard is ISO 14021 [109], which outlines requirements for self-declared environmental claims such as “compostable”, “degradable”, and “recyclable” and provides verification methods for related symbols [110]. EN 15343 [111] ensures the traceability of recycled plastics and calculates recycled content in products. EN 15347 [112] specifies the data suppliers must provide about plastic waste, including mandatory details such as mass, color, form, polymer types, and packaging, as well as optional data on properties like impact strength, additives, and contaminants.

Despite these efforts, global disparities in recycling protocols and regulatory frameworks present challenges [110,113]. Harmonizing standards internationally could foster collaboration, ensure material compatibility, and accelerate the adoption of recycled composites in various applications.

## 3. Methodology and Approach for Data Collection

This section outlines the methodology and approach for collecting data on the recycling of FRP composites through a systematic and comprehensive literature review. The primary aim is to identify and analyze scholarly publications that enhance the understanding of recycling techniques applied to composite materials. The Web of Science (WOS) academic database was used, with a combination of keywords such as “FRP” or “Fiber Reinforced Polymers” and “recycled” to ensure broad coverage of the research landscape. The WOS database was chosen for its comprehensive coverage of high-quality, peer-reviewed literature across diverse disciplines. The search was restricted to documents in English and published up to 2024, focusing on journal articles and reviews. A total of 1525 documents were retrieved. Using these papers, the software VOSviewer was employed to quantitatively assess the literature, applying network analysis techniques to identify key authors, countries, and research collaborations. This tool was selected for its ability to generate visualizations and map relationships between various research elements [114,115].

However, there are some limitations to the study. First, the data were collected exclusively from the Web of Science (WOS) database, which, while comprehensive, may not cover all relevant publications in other databases such as Scopus or Google Scholar. Additionally, the search was restricted to documents published in English up to 2024, which may exclude non-English language publications or more recent works not yet indexed.

## 4. Results of Bibliometric Analysis

### 4.1. Analysis of the Number of Publications over Time

**Figure 2:**
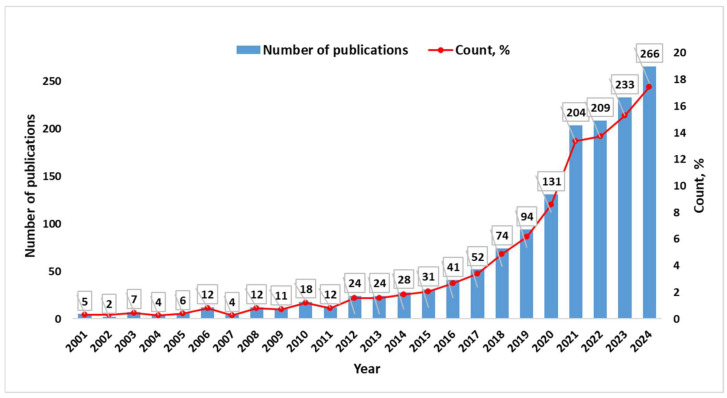
Number of publications by year.

Figure 2 illustrates the progressive increase in the number of publications over the years from 2001 to 2024, accompanied by a growing percentage trend that reflects heightened research activity. Initially, from 2001 to 2014, the growth in publications was gradual and steady. During this phase, the number of publications remained relatively low, with only slight fluctuations. This period can be described as one of modest development, where research in the field was still gaining momentum. However, a noticeable shift occurs from 2015 onwards, marking the beginning of a phase characterized by significant growth. As the years progress, there is a clear acceleration in the number of publications, with a pronounced rise evident after 2018. This upward trajectory becomes particularly striking in the most recent years, culminating in the highest recorded publication count in 2024. Simultaneously, the percentage growth also follows a steep curve, signaling an intensifying focus on the research area.

The analysis of document sources revealed the most influential journals in the field (Table 3). The *Construction and Building Materials* journal stands out with the highest number of publications (112), emphasizing its central role in advancing knowledge in this domain. Journals such as Polymers (62 publications), Composites Part B Engineering (49 publications), and the Journal of Cleaner Production (49 publications) indicate strong interdisciplinary research intersections, particularly in material science, sustainability, and engineering. Journals like Resources Conservation and Recycling (30 publications) and Sustainability (17 publications) reflect growing attention to environmentally conscious research, highlighting the importance of sustainable practices in construction and material development. Specialized journals such as Polymer Composites, Composite Structures, and the Journal of Reinforced Plastics and Composites underline advancements in composite materials, reinforcing their critical role in modern engineering and construction.

It can be observed that most journals are in the Q1 quartile, indicating their high academic standing, with some, like Composites Part B Engineering and Resources Conservation and Recycling, standing out for their prominent impact factors. While many high-impact journals, such as Construction and Building Materials, publish a large volume of articles, there are also journals with lower impact factors but still strong publication numbers.

### 4.2. Publication by Country

**Figure 3:**
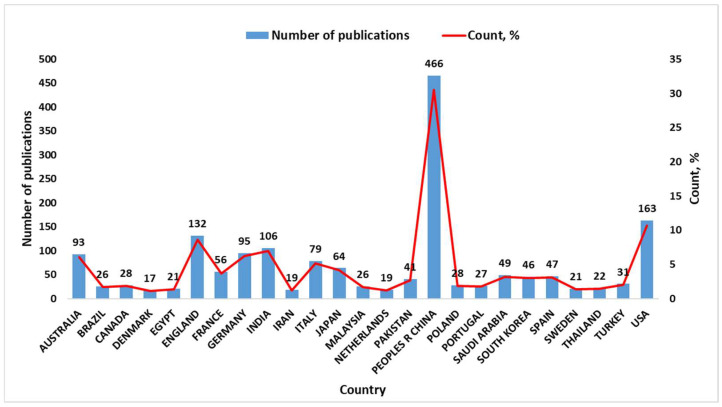
Number of publications by country.

Figure 3 illustrates the distribution of publications by country, represented by the number of publications (blue bars) and their corresponding percentage contribution (red line). It can be seen that China leads significantly with 466 publications, far exceeding other countries, reflecting its dominant role in this research field. The USA follows with 163 publications, highlighting its strong contribution, but is at a noticeably lower scale than China. Countries such as England (132), India (106), and Germany (95) show substantial but moderate contributions, indicating active research communities in these regions. Nations like Saudi Arabia (49), Republic of Korea (46), and Spain (47) demonstrate smaller yet notable contributions, reflecting diverse global participation in this research area.

### 4.3. Keyword Analysis

**Figure 4:**
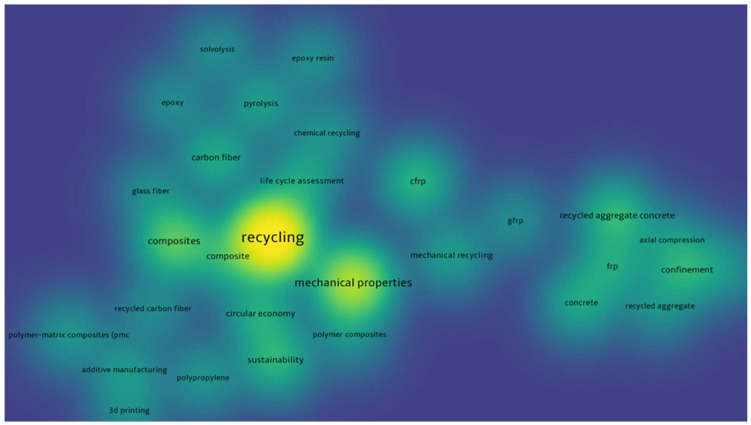
Keyword density visualization.

**Figure 5:**
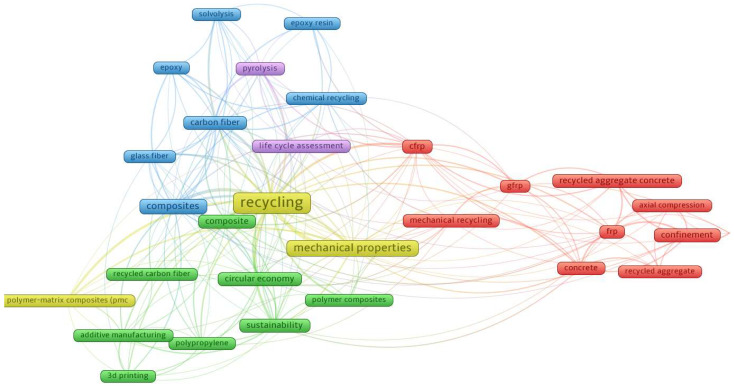
The keyword network.

VOSviewer was used to generate a density visualization of keyword recurrence in the selected documents. The minimum number of keyword occurrences was set to 20, and out of the 3822 keywords identified, 32 met this threshold. The resulting keyword density visualization is shown in Figure 4, while the network visualization is illustrated in Figure 5.

The heatmap from Figure 4 visually represents the relationships between research keywords in the field of recycling, with areas of higher brightness indicating more significant prominence and interconnectedness. At the center of the analysis lies the term “recycling”, reflecting its essential role in this research domain. Closely associated are themes such as “composites”, “mechanical properties”, and “sustainability”, which form the core of investigations into the reuse of materials and their performance in various applications.

The surrounding clusters highlight specific research directions. For instance, the focus on advanced materials is evident in the connections to “carbon fiber”, “glass fiber”, and processes like “chemical recycling” and “pyrolysis”. These terms suggest a growing interest in the recovery and reuse of composite materials. Similarly, terms such as “life cycle assessment” and “circular economy” emphasize the broader context of sustainability, reflecting efforts to evaluate the environmental impacts of recycling within a closed-loop framework.

On the other side of the map, the theme of mechanical properties is associated with construction materials, with terms such as “recycled aggregate concrete”, “frp” (fiber-reinforced polymer), and “axial compression”. This cluster suggests an emphasis on understanding the structural performance of recycled materials, especially in applications requiring high durability and strength. Emerging fields such as “additive manufacturing” and “3D printing” are also present, pointing toward innovative methods for integrating recycled materials into cutting-edge manufacturing processes.

As seen in Figure 5, in terms of clustered keywords, those related to “recycling” include “composites”, “carbon fiber”, “chemical recycling”, “life cycle assessment”, “circular economy”, and “sustainability”, highlighting a focus on sustainable practices, the use of composite materials, and the broader framework of recycling within a circular economy context. Overall, this map illustrates key research areas and their interconnections within recycling and materials science. The emphasis on sustainability, circular economy, and mechanical properties highlights ongoing efforts to innovate in recycling technologies and material applications. Additionally, emerging fields such as additive manufacturing and advanced composites suggest opportunities for future growth and exploration.

Emerging technologies and innovative methodologies are represented by clusters around keywords like “additive manufacturing”, “3D printing”, and “polymer–matrix composites”. These terms indicate growing interest in integrating recycled materials into cutting-edge production processes, showcasing the potential for combining sustainability with technological advancements. The inclusion of “sustainability” and “circular economy” ties these technological explorations to broader environmental and economic goals.

**Figure 6:**
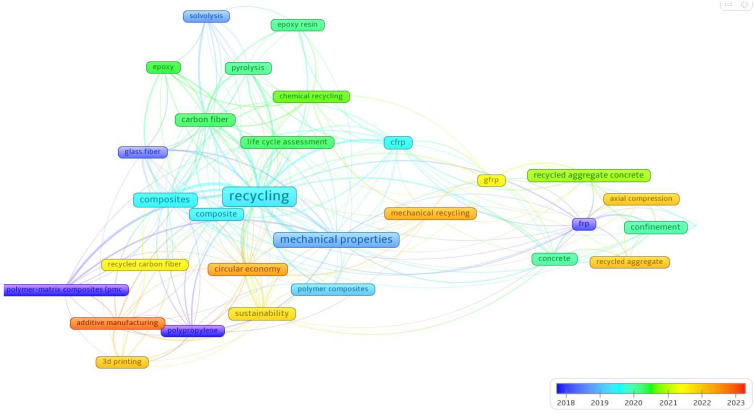
Temporal evolution of keywords related to recycled FRPs (2018–2023).

The map shown in Figure 6 incorporates a temporal dimension, as indicated by the color gradient ranging from blue to orange, which reflects the progression of research from 2018 to 2023. The temporal gradient reveals that research topics such as “3D printing”, “additive manufacturing”, and “circular economy” have gained prominence in recent years, as indicated by their orange hue. This suggests a shift toward more futuristic and application-oriented studies, while foundational themes like “composites” and “chemical recycling” maintain their relevance, as shown by their consistent connections throughout the network.

### 4.4. Co-Authorship Network

**Figure 7:**
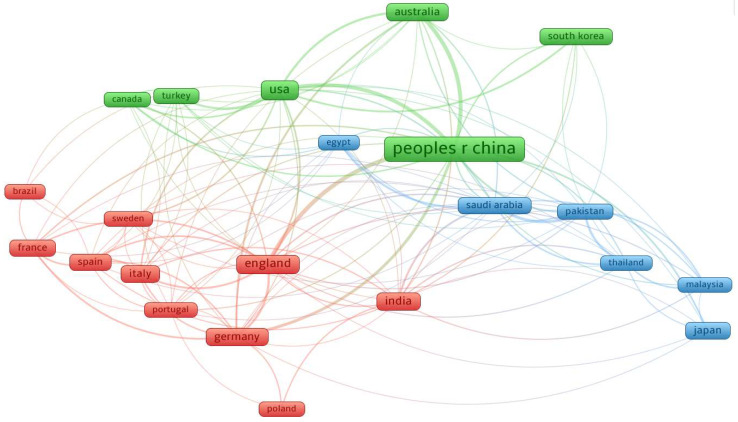
Network of country co-authorship.

This bibliometric network map presented in Figure 7 represents the collaboration and research connections between countries in the field under analysis.

At the center of the map, “People’s Republic of China” emerges as a dominant player, signified by its large, central node and extensive connections. The country’s position reflects its prominent role in global research efforts and its strong collaborations with other major contributors such as the USA, Australia, Republic of Korea, and Japan.

The USA, positioned prominently within its cluster, maintains strong connections to a wide range of countries, including Canada, Turkey, and Australia, as well as European nations such as England and Germany.

In the European cluster, countries such as England, Germany, France, and Italy form a closely connected group. This regional grouping, shown in red, highlights strong intra-European collaborations, while maintaining links to non-European countries, such as India and Brazil. England, in particular, appears to play a bridging role, connecting European research with other global networks.

India, situated within the red cluster but bridging into the broader network, signifies its active involvement in international research partnerships. Its strong links to countries like Saudi Arabia, Japan, and the USA illustrate its growing prominence in global collaborations.

Smaller clusters and connections reveal the involvement of countries like Saudi Arabia, Pakistan, and Thailand, often working with both regional and global leaders in the network. These nations reflect the diversification of research contributions from emerging economies and their increasing integration into global collaborations.

**Figure 8:**
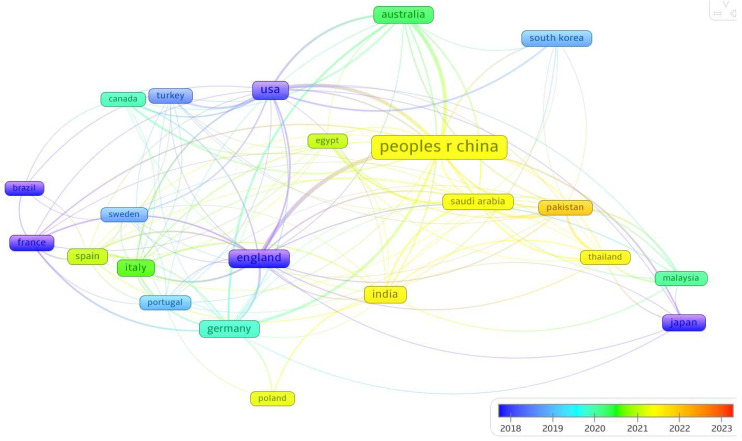
Temporal evolution of global co-authorship (2018–2023).

Figure 8 shows a bibliometric network map representing the global collaboration landscape, integrating a temporal gradient that reflects the evolution of partnerships from 2018 to 2023. The color-coded timeline, from blue to orange, highlights the progression and intensity of international research collaborations over time.

At the center of the network, “People’s Republic of China” emerges as the most prominent node, symbolizing its dominant role in global research output and collaboration. The extensive connections radiating from China link it to major research hubs, including the USA, England, and India, as well as other significant contributors such as Japan, Republic of Korea, and Australia.

The USA, marked by a strong presence, serves as a bridge connecting diverse regions. Its collaborations with countries such as Canada, Turkey, and England illustrate its influential role in facilitating cross-continental research. Similarly, England and Germany stand out within the European cluster, maintaining robust partnerships with both neighboring European countries—such as France, Italy, and Sweden—and global players like India and Brazil. These connections highlight Europe’s cohesive research ecosystem, bolstered by active international engagement.

India appears prominently linked to both traditional research leaders, such as the USA and England, and emerging contributors, such as Saudi Arabia and Pakistan. Its position signifies a growing influence in global research and an active role in fostering partnerships across regions. Japan and Republic of Korea, situated in the Asia–Pacific cluster, maintain strong ties with China and the USA, further emphasizing their contributions to regional and global collaborations.

The temporal gradient provides additional insights into the dynamics of these relationships. Emerging collaborations, marked by orange and yellow colors, reflect more recent research activities, particularly involving countries like Thailand, Saudi Arabia, and Pakistan. Conversely, established partnerships, shown in blue and green, represent longstanding connections that continue to underpin the research landscape.

## 5. Conclusions

This review has comprehensively examined the recycling of composite materials, with a particular focus on the mechanical properties of recycled FRPs and their implications for structural applications. Key findings include the variability in mechanical property retention based on the recycling method, with pyrolysis retaining up to 93% of tensile strength under optimal conditions, compared to reductions of 29% or more in mechanical recycling. The data underscore the potential of integrating recycled composites into sustainable design while identifying critical gaps in current practices.

Quantitative analyses revealed significant advancements in recycling technologies, with innovations such as hybrid composites enhancing material performance. In terms of environmental and economic impact, chemical recycling methods demonstrated lower CO_2_ emissions, but their scalability remains limited by high operational costs.

The bibliometric analysis reveals a significant rise in research activity on recycled composite materials, particularly FRPs, from 2001 to 2024. The number of publications increased sharply after 2015, with a peak in 2024, reflecting growing global interest in sustainable material solutions. Journals like Construction and Building Materials (112 publications) and Polymers (62 publications) emerged as key sources, demonstrating the interdisciplinary nature of the field.

China leads in publication output with 466 studies, followed by the USA and England, indicating strong research contributions globally. Keyword analysis highlighted “recycling”, “mechanical properties”, and “sustainability” as central themes, with recent trends focusing on innovative technologies like “3D printing” and “circular economy”.

This analysis underscores the rapid development of recycled composite research and the importance of global collaboration. Future efforts should focus on expanding partnerships and addressing research gaps to enhance the adoption of recycled materials in sustainable engineering practices.

Despite these advancements, challenges such as the lack of standardized recycling protocols and the high variability in material quality impede broader industrial adoption. Addressing these challenges requires interdisciplinary collaboration and robust lifecycle assessments to optimize both environmental and economic outcomes.

This review highlights the pressing need for innovative solutions to integrate recycled composites into mainstream applications, aligning with global sustainability goals. To further advance the field of recycled composite materials, future research should prioritize several key areas. These include the development of standardized recycling protocols to ensure consistent material quality, as well as the exploration of hybrid composites that combine recycled and virgin fibers to enhance mechanical performance. Additionally, expanding the application of advanced manufacturing technologies, such as 3D printing, can unlock new design possibilities for recycled composites.

## Figures and Tables

**Table 1:** Details on FRP recycling technologies.

Recycling Technology	Description	Advantages	Disadvantages	Reviewed Sources
Mechanical	Mechanical recycling is a conventional method for recycling thermoset matrix composites, where the material is reduced in size through grinding, cutting, milling, or shredding operations. Initially, the composite is cut into small pieces (50–100 mm) and manually cleared of inserts before being fed into a shredder. These pieces are then ground into smaller particles ranging from 10 mm to 50 μm. The resulting larger particles, which contain fibrous material, are typically used as filler in bulk molding compounds (BMCs), while the finer particles are suitable for sheet molding compounds (SMCs). The process involves key equipment, such as granulators or hammer mills, pulverizers for further crushing, and classifiers (e.g., cyclones or sieves) to separate the coarse fibrous and fine powdered products. Additionally, recycled GFRP aggregates, both coarse and fine, can be used in polyester-based mortar and concrete.	✓ The mechanical recycling process avoids the use of hazardous materials and effectively recovers both fibers and resin. ✓ Recycling carbon fibers (CF) requires significantly less energy—up to 100 times less—than the production of virgin carbon fibers. ✓ High efficiency and output rates. ✓ Scalable for industrial use. ✓ Minimal air or water pollution from gas emissions or chemicals. ✓ Cost-effective equipment requiring no skilled labor.	✓ Recycled products are limited to powders or short fibers, restricting their applications. ✓ Mechanical properties degrade, making them suitable only as fillers in new composites. ✓ The competitive advantage is questionable due to the availability of cheap calcium carbonate, a common filler in SMCs and BMCs. ✓ Energy-intensive recycling processes increase costs.	[18,46,58,59,60,61,62,63]
Thermal—fluidized bed	This involves burning the resin matrix in a hot, oxygen-rich flow, allowing for clean fiber recovery without char deposits. However, fiber length and strength degradation can occur during this process. The composite waste is first reduced to 25 mm and fed into a fluidized silica sand bed, which is heated to 450–550 °C using a hot air stream. Both the fibers and resin are carried by the stream, with the fibers separated by a cyclone and the resin fully oxidized in an afterburner, recovering energy as heat.	✓ No residual char remains on the fiber surface. ✓ Tolerance for contaminated materials. ✓ Recovery of energy.	✓ Strength degradation ranging from 25% to 50%. ✓ Fiber length degradation. ✓ Inability to recover material from resin.	[18,46,58,61,64,65,66,67,68]
Thermal—pyrolysis	Pyrolysis is a thermal recycling method that involves the decomposition of organic materials in the absence of oxygen. During this process, composite materials are exposed to high temperatures (450–750 °C), causing the matrix to break down into lower-weight molecules, while the fibers remain unaffected and can be recovered. The decomposition of the matrix produces oil, gases, and solid particles (char and fillers). The gases, primarily consisting of hydrogen, methane, carbon monoxide (CO), carbon dioxide (CO_2_), and other hydrocarbons, can be used for energy recovery. This process typically occurs in a static pyrolysis reactor under nitrogen.	✓ High retention of mechanical properties. ✓ Potential for recovering chemical feedstock from the resin. ✓ No use of chemical solvents.	✓ Potential for char deposition on the fiber surface. ✓ Sensitivity of recycled fiber properties to processing parameters. ✓ Generation of environmentally hazardous off-gases.	[18,46,58,65,69,70,71,72,73]
Chemical (solvolysis)	The chemical process of recycling composites is known as solvolysis. In this technique, the polymer matrix is degraded through exposure to a solvent. Solvolysis can be classified into two types: (a) solvolysis at lower temperatures and (b) solvolysis in supercritical fluids, depending on the temperature and state of the solvent. Additionally, solvolysis can be further categorized based on the type of solvent used, such as hydrolysis (using water), glycolysis (using glycols), and acid digestion (using acid).	✓ Excellent retention of mechanical properties and fiber length. ✓ High potential for material recovery from the resin. ✓ Use of low-risk solvents, such as alcohols, glycols, and supercritical water. ✓ Recovery of clean fibers with full length.	✓ Sensitivity to contaminants, such as metal inserts, which must be removed prior to the recycling process. ✓ Low efficiency and high cost. ✓ High energy consumption due to the high temperature and high pressure. ✓ Large amounts of solvents required.	[18,46,58,61,65,74,75,76,77,78]

**Table 2:** Recycling effects on the mechanical properties of FRPs.

Recycling Method	Effect on Mechanical Properties	Source Paper
Mechanical	A decrease of 29% in tensile strength, 23% in Young’s modulus, 28% in flexural strength, and 24% in flexural modulus was demonstrated.	[63]
	The bending modulus of coarse and fine fibers increased by approximately 161% and 80%, respectively, compared to the resin alone. The coarse and fine samples showed reductions in strain at failure of approximately 32% and 45%, respectively. The fine sample exhibited a slight reduction in bending strength of about 14.7%.	[79]
	A decrease in flexural strength by 9%, an increase in impact strength by 7%, and a decrease in flexural modulus by 3% compared to the standard composite were demonstrated.	[80]
	The addition of carbon powder wastes (CPW) significantly enhanced the resin’s mechanical properties. With 10 wt.% and 20 wt.% CPW, the flexural strength increased by 14% and 30%, the modulus of elasticity by 10% and 30%, and impact strength by 3% and 28%, respectively. The compressive strength improved by 6% and nearly 20% with 10 wt.% and 20 wt.% CPW, respectively.	[81]
Thermal	A decrease of 19% in flexural strength and an increase of 3.6% in flexural modulus were demonstrated.	[82]
	An increase in tensile strength from 140 MPa to 149 MPa (6.4%) and an increase in Young’s modulus from 3.9 GPa to 4.1 GPa (5.1%) were demonstrated.	[83]
	The best retention of carbon fiber characteristics (93% of virgin tensile strength) was obtained when composite waste was pyrolyzed and oxidized at 500 °C. In comparison, when the composite waste was pyrolyzed at 350 °C and oxidized at 700 °C, it retained only 26% of virgin carbon fiber tensile strength.	[84]
	When CFRP decomposed at 600 °C, the tensile strength decreased by about 50%, as the oxygen concentration increased from 5% to 20%. At 650 °C, the tensile modulus decreased in air, while the tensile strength stabilized with high oxygen concentrations. At 650 °C and 5% O_2_, the tensile properties remained stable, with the best retention of tensile strength (about 80%) observed at 650 °C with 5% O_2_ for 45 min.	[85]
	The tensile strength of recovered E-glass fibers was reduced by up to 50%.	[64]
	Flexural and Young’s moduli remained unchanged, but flexural and tensile strength decreased when over 50% of the virgin reinforcement was replaced by fibers recovered at 450 °C.	[86]
Chemical	A 350 °C hydrolysis temperature reduced mechanical properties by 60%, while 300 °C caused a 50% reduction.	[87]
	A reduction of 10% in tensile strength was found.	[88]
	The tensile strength of all recovered fibers was similar to that of the virgin fiber after sizing removal.	[77]
	The recycled glass fibers (GFs) retained approximately 92.7% tensile strength, 99.0% Young’s modulus, and 96.2% strain-to-failure compared to virgin GFs.	[89]
	The tensile strength of the recovered CFs was over 95% of that of the virgin fibers.	[75]
	The CFs retained approximately 92% tensile strength and 94% strain-to-failure, compared to the original CFs.	[90]

**Table 3:** Top document sources.

Journal	Journal Impact Factor (2024)	Journal Quartile 2024	Number of Publications
*Construction and Building Materials*	7.4	Q1	112
Polymers	4.7	Q1	62
Composites Part B Engineering	12.7	Q1	49
Journal of Cleaner Production	9.8	Q1	49
Composite Structures	6.3	Q1	45
Polymer Composites	4.8	Q1	40
Materials	3.1	Q1	39
Journal of Building Engineering	6.7	Q1	31
Resources Conservation and Recycling	11.2	Q1	30
Structures	3.9	Q1	29
Composites Part A Applied Science and Manufacturing	8.1	Q1	26
Engineering Structures	5.6	Q1	26
Polymer Degradation and Stability	6.3	Q1	26
Composites Science and Technology	8.3	Q1	24
Journal of Applied Polymer Science	2.7	Q2	24
Journal of Reinforced Plastics and Composites	2.3	Q3	24
Journal of Composites For Construction	2.9	Q2	23
ACS Sustainable Chemistry Engineering	7.1	Q1	20
Journal of Composite Materials	2.3	Q3	17
Sustainability	3.3	Q2	17

## Data Availability

The data used are available through academic databases.
